# Deterioration of neuronal primary cilia in Alzheimer’s disease

**DOI:** 10.1002/alz.093226

**Published:** 2025-01-03

**Authors:** Emiko Miller, Peter Bambakidis, Niklas Reitsch, Phoebe Templin, Edwin Vázquez‐Rosa, Zea Bud, Youngmin Yu, Hisashi Fujioka, Feixiong Cheng, Marina Bykova, Yeojung Koh, Preethy Sridharan, Coral Cintrón‐Pérez, Kathryn Franke, Andrew A. Pieper

**Affiliations:** ^1^ Case Western Reserve University, Cleveland, OH USA; ^2^ University Hospitals, Cleveland, OH USA; ^3^ University Hospitals Cleveland Medical Center, Cleveland, OH USA; ^4^ Louis Stokes Cleveland VA Medical Center, Cleveland, OH USA; ^5^ University of Toledo, Toledo, OH USA; ^6^ Cleveland Clinic, Cleveland, OH USA; ^7^ Cheveland Clinic Genome Center, Cleveland, OH USA; ^8^ Biological, Geological, and Environmental Sciences, Cleveland State University, Cleveland, OH USA; ^9^ Lerner Research Institute, Cleveland Clinic Foundation, Cleveland, OH USA; ^10^ Louis Stokes VA Medical Center, Cleveland, OH USA

## Abstract

**Background:**

Emerging evidence links Alzheimer’s disease (AD) to dysfunction of the primary cilium, a historically overlooked organelle that serves as the neuron’s antenna. All neurons harbor a single primary cilium that projects from the membrane to sense changes in the extracellular environment. Primary cilia dysfunction leads to a group of diseases called ‘ciliopathies’, which are associated with reduced hippocampal and cortical mass, as well as neurocognitive impairment. Ciliopathies place individuals at higher risk for dementia, and genetic defects in primary cilia proteins in mice lead to a phenotype resembling AD.

**Method:**

To establish whether primary cilia are disrupted in AD, we conducted unbiased analysis of differentially expressed genes (DEGs) in postmortem brains from AD subjects, compared to age‐matched non‐AD controls. Using the DAVID bioinformatics platform, we performed a gene ontology analysis on the significantly changed genes related to the primary cilia. Additionally, we performed immunohistochemical and electron microscopy analysis of primary cilia in the brains of 5xFAD mice, a mouse model of AD. Primary cilia were visualized with antibodies to adenylate cyclase 3 (AC3).

**Result:**

Our systematic survey of the published literature in AD revealed very few studies to date of cilia, compared to other organelles. Our analysis of DEGs in postmortem human AD brains revealed a high prevalence of DEGs intricately linked to processes that maintain primary cilia structure and function. Furthermore, the proportion of DEGs related to the primary cilia was comparable to that observed in other heavily implicated organelles in AD, such as mitochondria. Additionally, we found that the gene *ADCY3*, which encodes for primary cilia‐specific adenylate cyclase 3 (AC3), is significantly downregulated in human AD brains. Primary cilia are dependent on AC3, and loss of functional AC3 in primary cilia reveals consequences reminiscent of symptoms associated with AD. We observed reduced levels of AC3 throughout the hippocampus of 5xFAD mice. Additionally, electron microscopy analysis of primary cilia revealed shortened primary cilia length in advanced stages of disease in 5xFAD mice.

**Conclusion:**

AC3 loss and primary cilia deterioration may drive progression of AD pathology and provide a basis for development of a new neuroprotective therapy for AD.

